# Loss of CEACAM1 is associated with poor prognosis and peritoneal dissemination of patients with gastric cancer

**DOI:** 10.1038/s41598-019-49230-w

**Published:** 2019-09-03

**Authors:** Akihiro Takeuchi, Shozo Yokoyama, Mikihito Nakamori, Masaki Nakamura, Toshiyasu Ojima, Shunsuke Yamaguchi, Yasuyuki Mitani, John E. Shively, Hiroki Yamaue

**Affiliations:** 1 Second Department of Surgery, School of Medicine, Wakayama Medical University, Wakayama, 641-8510 Japan; 2 Department of Molecular Imaging & Therapy, Beckman Research Institute of the City of Hope, Duarte, CA 91010 USA

**Keywords:** Prognostic markers, Tumour biomarkers

## Abstract

CEACAM1 is associated with malignant potential of various cancers. The current study aims to clarify the association between carcinoembryonic antigen-related cell adhesion molecule 1 (CEACAM1) expression and malignant potential of gastric cancer and to address whether CEACAM1 cytoplasmic domain isoform balance modulates the properties of gastric cancer cells. Immunohistochemical analyses for CEACAM1 were performed in 235 patients with gastric cancer who underwent surgery. Risk factors for overall survival and peritoneal metastasis were calculated based on CEACAM1 expression in the gastric cancer tissue. Patients with CEACAM1 long (CEACAM1-L) or short (CEACAM1-S) cytoplasmic isoform dominance were compared with patients with null CEACAM1 expression in terms of overall survival. CEACAM1 transfected or knockdown gastric cancer cell line, NUGC3 and MKN7 cells, were examined by invasion assay and three dimensional (3D) culture, in order to clarify whether CEACAM1 modulate invasion, lumen formation and tumor growth of gastric cancer cells. Multivariate analysis demonstrated that gastric cancer without CEACAM1 is an independent prognostic factor and a risk factor for peritoneal dissemination. Patients with CEACAM1-S dominance had better prognosis than those with CEACAM1-L. CEACAM1-4L overexpression induced less invasion, more lumen formation, and less tumor growth of NUGC3 cells. CEACAM1-4S overexpression had less invasion and more lumen formations, but not less tumor growth. Knockdown of CEACAM1 expression had less invasion, but not less lumen formations of MKN7 cells. Loss of CEACAM1 is associated with poor prognosis and peritoneal dissemination of patients with gastric cancer. Expression of CEACAM1 in gastric cancer cells modulates invasiveness, lumen formation, and tumor growth.

## Introduction

Carcinoembryonic antigen related cell adhesion molecule 1 (CEACAM1) belonging to the immunoglobulin superfamily and the carcinoembryonic antigen (CEA) family is a transmembrane protein and cell–cell adhesion molecule. It was also known as CD66a, and biliary glycoprotein^1^. CEACAM1 is associated with various cancers, and its expression level and function is different among them. It has reported that CEACAM1 is downregulated in breast^2^, prostate^3^, and endometrial^4^ cancer tissues. CEACAM1 seems to be a tumor suppressor. Whereas, CEACAM1 overexpression is related to malignant grade of cutaneous malignant melanoma^5^, lung adenocarcinoma^6^, advanced colon cancer^7^ and hepatocellular carcinoma^8^. Whether CEACAM1 is a suppressor or promoter for malignant phenotype of cancer cells still remains controversial.

In colorectal cancer and hepatocellular carcinoma (HCC), we have reported that CEACAM1 is associated with invasion, metastasis and poor prognosis^7–9^. CEACAM1 expression re-expressed at the invasion front of clinical colorectal cancer tissue^7^. CEACAM1 long cytoplasmic domain isoform (CEACAM1-L) dominance is correlated to lymph node and distant metastasis, and poor prognosis^7^. CEACAM1 long cytoplasmic domain isoform (CEACAM1-4L) promotes colorectal cancer cell invasion and migration^7^. In colorectal cancer liver metastasis, enhanced CEACAM1 short cytoplasmic domain isoform (CEACAM1-S) on colorectal cancer cells with overexpressed CEACAM1-L was associated with recurrence after curative hepatectomy^9^. We also reported that CEACAM1-L is correlated with recurrence-free and disease-specific survival of patients with HCC. CEACAM1-4L enhanced invasion and migration of HCC cells^8^. In gastric cancer, there is a possibility that similar changes, such as colorectal cancer and HCC, may occur by expression and cytoplasmic domain isoform balance of CEACAM1. In gastric cancer, it has been reported that high levels of CEACAM1 are associated with angiogenesis^10,11^, and that co-expression of CEACAM1 and TGF beta may be correlated with angiogenesis^12^. However, the association between CEACAM1 and clinicopathological features of patients with gastric cancer requires investigation. The role of CEACAM1 cytoplasmic isoform balance is also unclear.

In the present study, we investigated the association between CEACAM1 expression in gastric cancer tissues and clinicopathological features of patients with gastric cancer, including prognosis and peritoneal dissemination which is an important prognostic factor for patients with gastric cancer. We then examined implications of CEACAM1 cytoplasmic domain isoform balance for patients with gastric cancer. Moreover, we explore the notion that enhanced CEACAM1 cytoplasmic isoform (CEACAM1-4L or CEACAM1-4S) modulates malignant properties such as invasion, lumen formation and tumor growth of gastric cell line.

## Results

### Relationship between CEACAM1 expression and malignant potential of gastric cancer after gastrectomy

**Figure 1:**
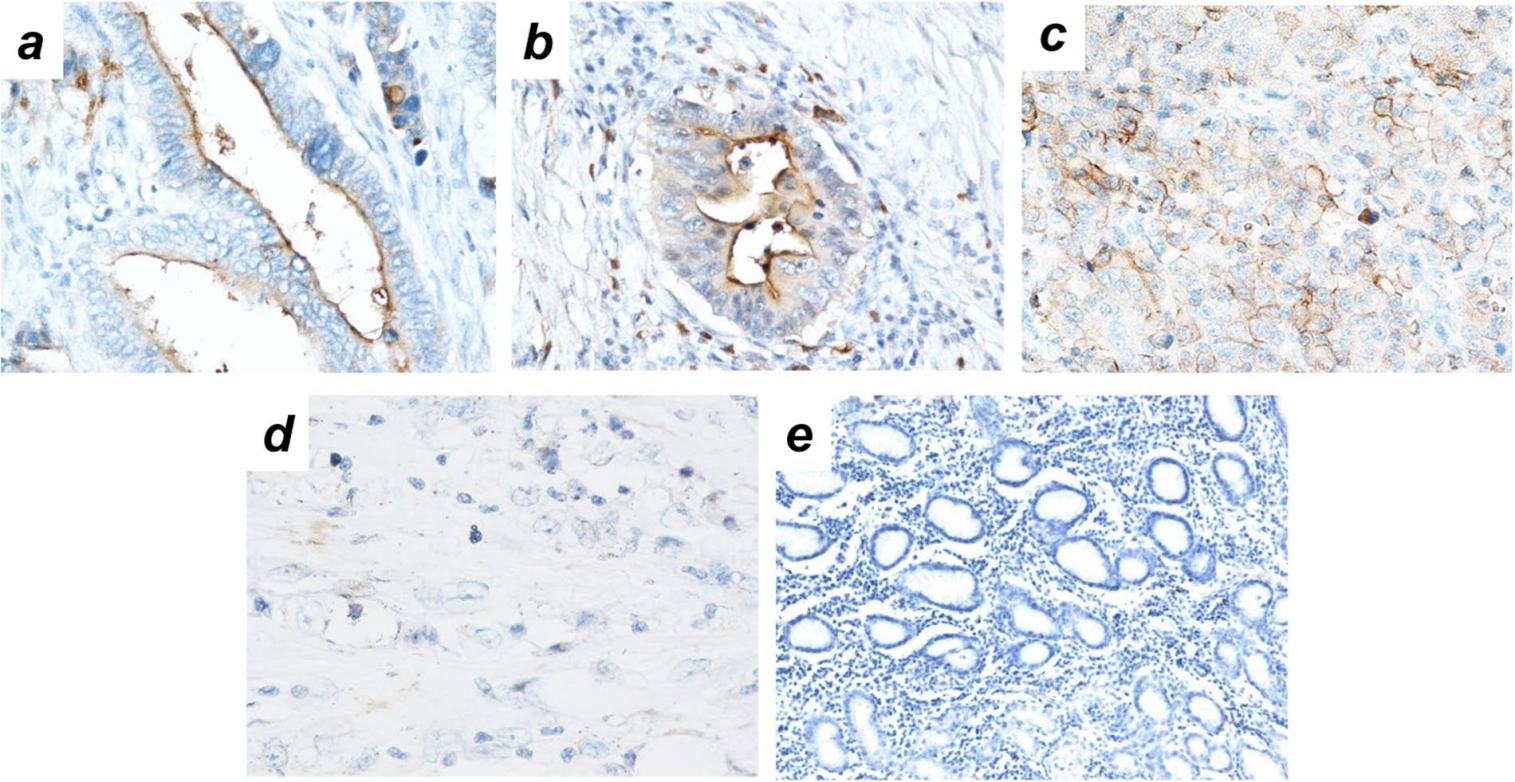
Expression pattern of CEACAM1 in gastric cancer. Immunohistochemical staining of CEACAM1 (×100 magnification): (**a**) well differentiated (tub1), (**b**) moderately differentiated (tub2), (**c**) Poorly differentiated (por), (**d**) Signer-ring cell carcinoma (sig) (**e**) Normal gastric mucosa as a negative control.

Among 235 patients, 157 (66.8%) exhibited CEACAM1 positive staining of the gastric cancer cells. CEACAM1 stained lumen surface for adenocarcinoma that performed lumen, whereas CEACAM1 stained cancer cell membrane for adenocarcinoma that does not lumen formation (Fig. 1). We divided cases into two groups in presence or absence of expression of CEACAM1 in the deepest part of the tumor. Comparison of clinicopathologic variables between the CEACAM1 expression groups and the non CEACAM1-expression groups indicated that CEACAM1 expression was significantly correlated with diffuse type gastric cancer (*p* < 0.001) and venous permeation (*p* = 0.022) (Table 1).

**Table 1:** Clinicopathological variables for CEACAM1-expression and non CEACAM1-expression groups.

Variable	Non CEACAM1-expression N = 78	CEACAM1-expression N = 157	*p*-value
Age (Median, Range)	66 (23–90)	69 (37–89)	0.059
Gender (male/female)	51/27	119/38	0.121
Differentiation (diffuse/intestinal)	56/22	42/115	<0.001
Depth (T1,T2/T3,T4)	49/29	96/61	0.887
Stage (I,II/III)	60/18	128/29	0.489
Lymph node metastasis (+/−)	32/46	62/85	0.888
Lymphatic permeation (+/−)	38/40	87/70	0.405
Venus permeation (+/−)	21/57	67/90	0.022

**Figure 2:**
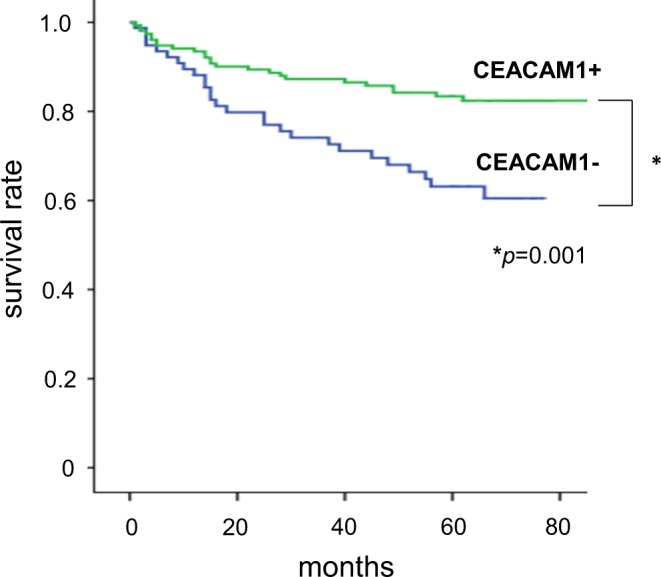
Kaplan-Meier estimates of survival. Comparison of CEACAM1 expression groups and non CEACAM1-expression groups.

Univariate analysis revealed that depth (>T3), lymph node metastasis, lymphatic permeation, venous permeation and non CEACAM1-expression were significant predictive factors for overall survival. On the basis of multivariate analysis, depth (>T3), lymph node metastasis and non CEACAM1-expression were selected as independent predictive factors for overall survival (Table 2). The Kaplan-Meier method for the overall survival analysis showed that non CEACAM1-expression group was significantly associated with a shorter survival time (Fig. 2).

**Table 2:** Univariate and multivariate analyses of overall survival after gastrectomy 235 patients.

Variable	Univariate analysis			Multivariable analysis		
	HR	95% CI	*p*-value	HR	95% CI	*p*-value
Age ( ≧ 75 years)	1.003	0.468–1.597	0.642			
Gender (male)	1.387	0.378–1.373	0.320			
Differentiation (diffuse)	2.110	1.127–3.663	0.008	1.564	0.648–2.475	0.421
Depth (T3,T4)	12.968	5.965–27.028	<0.001	10.879	1.842–11.012	0.001
Lymphatic permeation	8.401	3.584–19.696	<0.001	2.195	0.778–6.072	0.138
Venous permeation	2.987	1.870–4.772	<0.001	1.528	0.803–2.627	0.216
Lymph nodes metastasis	11.125	5.224–23.694	<0.001	4.229	1.048–7.079	0.04
non CEACAM1-expression	2.427	1.408–4.184	0.025	3.472	1.508–8.00	0.03

HR, hazard ratio; CI, confidential interval.

### Relationship between CEACAM1 expression and peritoneal dissemination of gastric cancer

The peritoneal dissemination of the gastric cancer is a fatal recurrent pattern for gastric cancer patients. The association between CEACAM1 expression and peritoneal dissemination was evaluated. Univariate analysis revealed that depth (>T3), lymph node metastasis, lymphatic permeation, venous permeation and non CEACAM1-expression were significant risk factors for peritoneal dissemination. On the basis of multivariate analysis, depth (>T3) and non CEACAM1-expression were selected as independent risk factors for peritoneal dissemination (Table 3).

**Table 3:** Univariate and multivariate analyses of peritoneal dissemination after gastrectomy 235 patients.

Variable	Univariate analysis			Multivariable analysis		
	HR	95% CI	*p*-value	HR	95% CI	*p*-value
Age ( ≧ 75 years)	1.203	0.504–2.867	0.677			
Gender (male)	0.955	0.415–2.197	0.914			
Differentiation (diffuse)	2.915	1.300–6.536	0.009	1.582	0.582–4.310	0.369
Depth (T3, T4)	56.534	7.648–417.879	<0.001	29.859	3.556–250.710	0.002
Lymphatic permeation	6.251	2.150–18.176	0.001	1.219	0.333–4.453	0.765
Venous permeation	3.051	1.583–5.879	0.001	1.599	0.705–3.626	0.261
Lymph nodes metastasis	11.772	4.040–34.303	<0.001	2.178	0.593–8.003	0.241
non CEACAM1-expression	3.145	1.443–6.857	0.004	3.711	1.253–10.995	0.018

HR, hazard ratio; CI, confidential interval.

### Relationship between CEACAM1 isoform balance and malignant potential, poor survival of gastric cancer after gastrectomy

**Figure 3:**
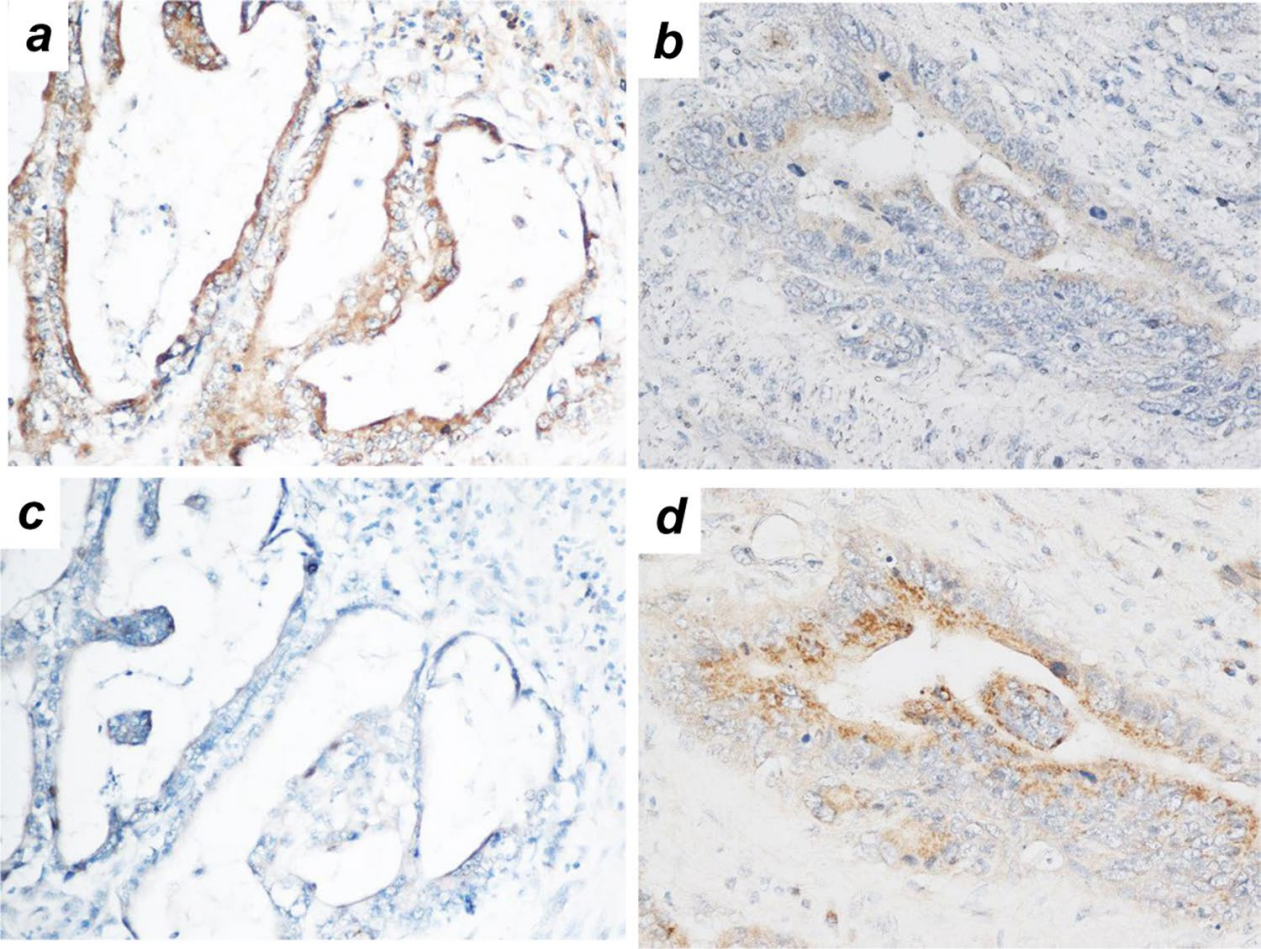
Expression pattern of CEACAM1 cytoplasmic isoforms. Immunohistochemical staining CEACAM1-L (**a**,**b**) and CEACAM1-S (**c**,**d**). (**a)** and (**c**), (**b**) and (**d**) are the same cases, respectively, (×400 magnification).

**Figure 4:**
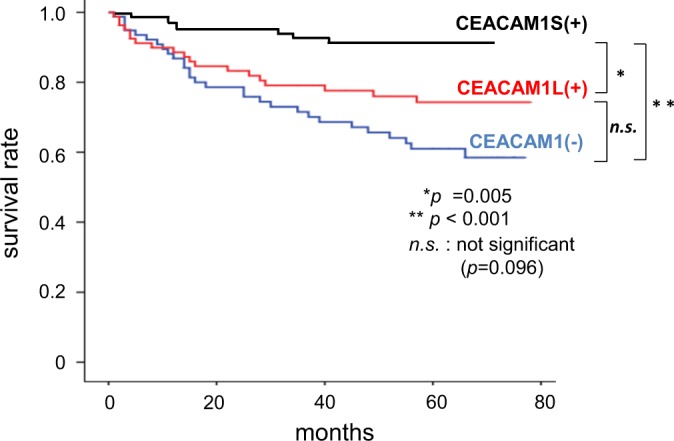
Kaplan-Meier estimates of survival. Survival rate of non CEACAM1-expresssion groups,CEACAM1-L dominant expression groups and CEACAM1-S dominant expression groups. n.s.; not significance.

Within CEACAM1 positive samples, CEACAM1-4L-dominant expression was observed in 81 patients (51.6%), CEACAM1-4S-dominant expression was observed in 76 patients (48.4%) (Fig. 3). Comparison of clinicopathologic variables between the CEACAM1-4L, CEACAM1-4S dominant expression groups showed that dominant expression of CEACAM1-4L was significantly correlated with diffuse type gastric cancer (*p* = 0.019) and lymph node metastasis (*p* = 0.022) (Table 4). The Kaplan-Meier method for the overall survival analysis showed that CEACAM1 4L-dominant group was significantly associated with a shorter survival time than CEACAM1-4S dominant group (*p* = 0.005). CEACAM1-4S dominant group also had good prognosis in comparison with non CEACAM1-expression group (Fig. 4).

**Table 4:** Clinicopathologic variables for CEACAM1-L dominant and CEACAM1-S dominant expression Groups

Variable	CEACAM1-L dominant expression n = 81	CEACAM1-S dominant expression n = 76	*p*-value
Age (Median, Range)	68 (38–89)	69 (37–88)	0.998
Gender (male/female)	56/25	63/13	0.121
Differentiation (diffuse/intestinal)	29/52	13/63	0.011
Depth (T1,T2/T3,T4)	48/33	51/25	0.887
Stage (I,II/III)	62/19	66/10	0.489
Lymph node metastasis (+/−)	39/42	23/53	0.888
Lymphatic permeation (+/−)	48/33	39/37	0.405
Venus permeation (+/−)	39/42	28/48	0.022

### CEACAM1 expression and invasion of gastric cancer cells

**Figure 5:**
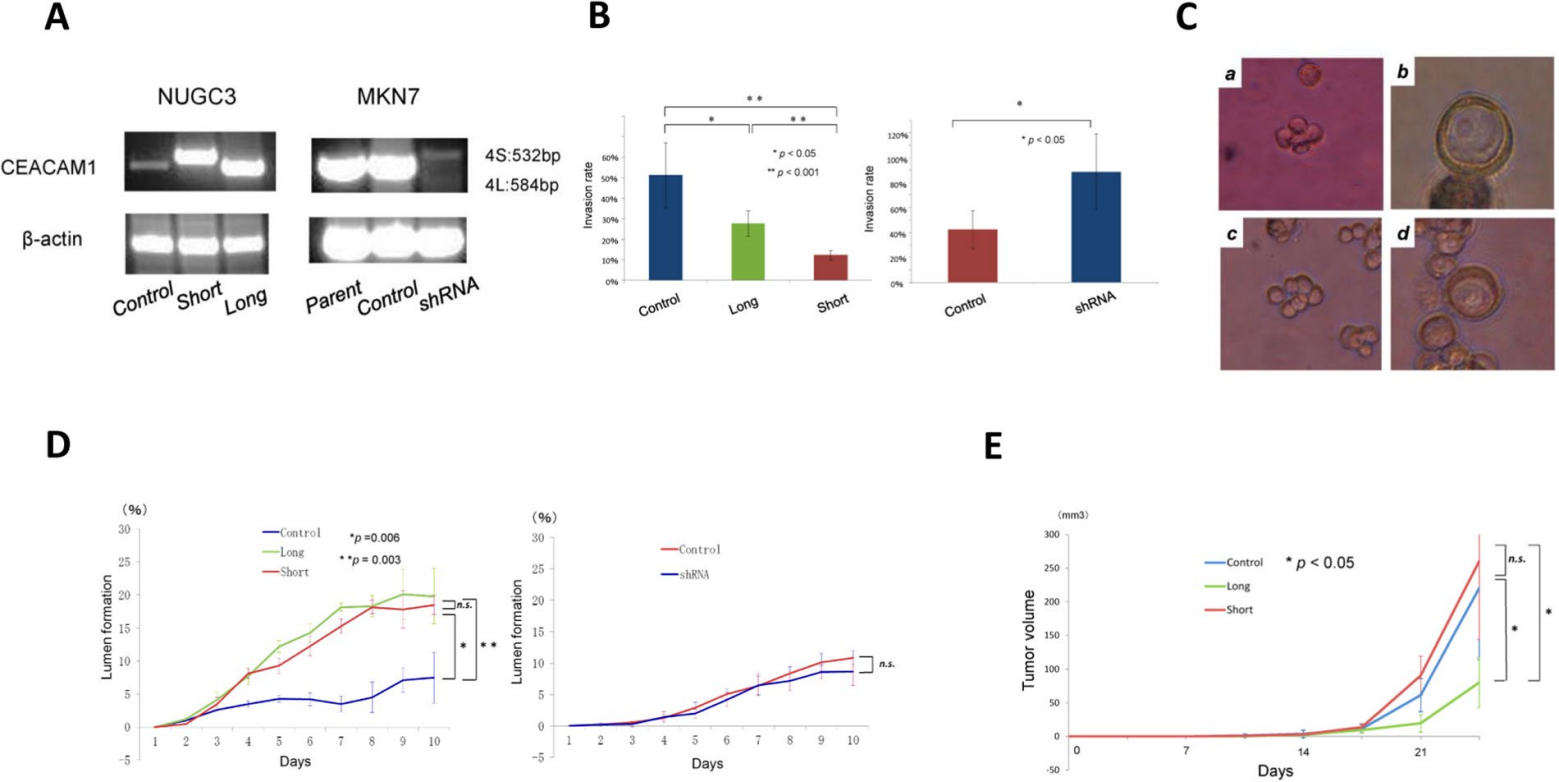
Effects of transfection with CEACAM1-4L/4S on invasion, lumen formation and tumor growth. Control; CEACAM1 vector control transfected NUGC3 (undifferebtiated type) and MKN7 (differentiated type) cells. Long; CEACAM1-4L transfected NUGC3 cells.Short; CEACAM1-4S transfected NUGC3 cells, shRNA; shRNA targeted to CEACAM1 transfected MKN7 cells. (**A**) RT-PCR analysis of NUGC3 cells with transfected vector control, CEACAM1-4L, CEACAN1-4S, and of MKN7 cells with transfected shRNA targeted to CEACAM. (**B**) *In vitro* invasion assay using Matrigel invasion chanbers. (**C**) Representative images of gastric cancer lumen formation in Matrigel 3D culture. *a*: no lumen formation, NUGC3 cells; *b*: lumen formation, NUGC3 cells; *c*: no lumen formation, MKN7 cells; *d*: lumen formation, MKN7cells. (**D**) Lumen formation in NUGC3 and MKN7 cells in 3D culture. n.s.; not significance. (**E**) *In vivo*, the subcutaneous tumor model with NOD/SCID mice. n.s.; not significance.

To evaluate the effect of CEACAM1 expression on invasion of gastric cancer cells, we performed invasion assay for CEACAM1-4L or CEACAM1-4S transfected NUGC3 cells and CEACAM1 knockdown MKN7 cells. In comparison with vector control, CEACAM1-4L and CEACAM1-4S both significantly suppressed invasion of NUGC3 cells. CEACAM1-4S inhibited invasion of NUGC3 cells more than CEACAM1-4L. CEACAM1 shRNA suppressed invasion of MKN7 cells (Fig. 5B).

### CEACAM1 expression and lumen formation of gastric cancer

In immunohistochemistry, most cases without CEACAM1 expression were found to be diffuse type gastric cancer. It was therefore speculated that CEACAM1 may be involved in lumen formation of gastric cancer cells. In order to evaluate the association between CEACAM1 expression and lumen formation, we observed morphological changes of Vector CEACAM1-4L or CEACAM1-4S transfected NUGC3 cells in 3D culture. NUGC3 cells with vector control significantly had lower lumen formation rate than those with CEACAM1-4L and CEACAM1-4S. The significant difference was absent between CEACAM1-4L and CEACAM1-4S (Fig. 5C,D). CEACAM1 shRNA did not suppress lumen formation of MKN7 cells (Fig. 5C,D).

### CEACAM1 expression and *in vivo* tumor growth

In the subcutaneous tumor model with NOD SCID mice, CEACAM1-4L transfected NUGC3 cells was more suppressed tumor growth than those with CEACAM1-4S or vector control. Conversely, there is no significant difference between CEACAM1-4S and vector control transfected NUGC3 cells in terms of tumor growth (Fig. 5E).

## Discussion

In the present study there was indication that loss of CEACAM1 expression in gastric cancer was an independent prognostic factor and independent risk factor of peritoneal dissemination. Moreover, CEACAM1-S dominance group had indication of better prognosis than CEACAM1-L dominance group. Overall survival of CEACAM1-L dominance group had no significant difference in those of loss of CEACAM1 group (Fig. 4). Poor prognosis conferred by CEACAM1-L dominance seems to be reconciled with better prognosis by CEACAM1-S dominance, leading to favorable overall survival conferred by whole CEACAM1 expression group (Fig. 2). To the best of our knowledge, this is the first report of the association between CEACAM1 expression and clinicopathological features.

It seems that CEACAM1 expression in gastric cancer cells can modulate tumor growth, invasion and lumen formation. The present study showed that both CEACAM1-4L and CEACAM1-4S suppressed invasion of NUGC3 cells. CEACAM1-4S suppressed more invasion than CEACAM1-4L. With respect to lumen formation, both CEACAM1-4L and CEACAM1-4S promoted lumen formation of NUGC3 cells. However, knockdown of CEACAM1 in MKN7 cells did not significantly suppress lumen formation. It is speculated that not only CEACAM1 induce lumen formation, but also various molecules would be involved in lumen formation. Regarding tumor growth, only CEACAM1-4L, but not short isoform, suppressed tumor growth of NUGC3 cells. Two cytoplasmic domain isoforms provoke different effects on gastric cancer cells. CEACAM1 is also reportedly associated with microenvironment^13,14^ and chemotherapy^15^. Further examinations are required to address the effect of CEACAM1 expression on gastric cancer cells.

In the clinical setting, Loss of CEACAM1 had worst survival among them. On the other hand, CEACAM1-S dominance group indicated best survival rate. CEACAM1-L dominance group shows poorer overall survival and more diffused phenotype than CEACAM1-S dominance group. *In vitro* studies were conducted to clarify why CEACAM1 is associated with the clinical observation. NUGC3 cells with loss of CEACAM1 had most invasiveness, worst lumen formation, and more tumor growth. It is clear that loss of CEACAM1 expression is the highest malignant grade. On the other hand, CEACAM1-4S induced suppression of invasion, more lumen formation, and more tumor growth. CEACAM1-4S attenuated malignant potential of gastric cancer cells except tumor growth. How about CEACAM1-4L. The differences between CEACAM1-4S and -4L were invasion and tumor growth. CEACAM1-4L induced more invasiveness and less tumor growth than CEACAM1-4S. Invasion of cancer cells is more important factor for malignant phenotype than tumor growth. We have already reported that CEACAM1-4L promotes invasiveness of colorectal cancer and hepatocellular carcinoma, resulting in that CEACAM1-L dominant expression is associated with poor survival. Therefore, the reason why CEACAM1-L shows poorer overall survival than CEACAM1-S may be due to CEACAM1-L induced invasiveness. Table 4 showed that CEACAM1-L dominance is more associated with diffuse subtype of gastric cancer. Our 3D culture experiments showed around 20% lumen formation of gastric cancer cells. Other 80% cells did not form lumen. Therefore, more invasive property of CEACAM1-L expressing cells, but not lumen formation, may reflect histological phenotype.

We have so far reported that CEACAM1 cytoplasmic domain balance, long cytoplasmic domain dominance, promoted migration, invasion, metastasis and poor prognosis. The present study demonstrated that CEACAM1 expression in gastric cancer cells can lead to tumor suppression, less invasion, more lumen formation, less peritoneal dissemination and better prognosis. Whether CEACAM1 is a good or bad molecule for cancer, however, remains controversial. CEACAM1 was formally thought to be a tumor suppressor, but its expression is downregulated in cancer tissues derived from colon^16^, breast^2^, prostate^3^ and endometrium^4^. In the current report the tumor suppressive function of CEACAM1 is consistent with this data. It is speculated that if the cancer cells are without CEACAM1 expression, enhanced CEACAM1 expression provokes less malignant phenotype of cancer including lumen formation^13,17–20^. On the other hand, we have reported that if the cancer cells constantly express CEACAM1 such as colorectal cancer and HCC, CEACAM1-L dominant balance promotes malignant phenotypes including invasion, metastasis and poor prognosis^7–9^.

We have argued that there is a difference between organs. Normal gastric mucosa does not express CEACAM1^12^, but non-neoplastic hepatocytes express CEACAM1^21^, and CEACAM1-L accounts for almost 15–20% of total CEACAM1 expression in normal colonic epithelial cells^22^. Physiological expression of CEACAM1 differs by organ. It is therefore conceivable that the role of expression and cytoplasmic domain isoform balance of CEACAM1 is already different between cells in the stomach, the colon, the rectum and the liver. The majority of colorectal cancer and HCC express CEACAM1 and it is rare that colorectal cancer and HCC have no expression of CEACAM1. In the background that cancer cells basically express CEACAM1, cancer cells modulate CEACAM1 cytoplasmic domain dominance, leading to promotion of malignant phenotypes such as motility, invasion, metastasis and prognosis. On the other hand, in cancer cells without CEACAM1 expression, such as gastric, prostate, and breast cancer, CEACAM1-L can function as a tumor suppressor. In general, CEACAM1-positive cancer such as colorectal cancer and HCC is not aggressive and has predictable prognosis, whereas CEACAM1-negative cancer, such as gastric cancer, is aggressive and has unpredictable prognosis. Based on this idea, CEACAM1 basically works as tumor suppressor for cancer. In more advanced stages, CEACAM1 expression and cytoplasmic domain isoform balance are involved in promoting motility and invasion of cancer cells.

The present study indicates that loss of CEACAM1 is associated with poor prognosis and peritoneal dissemination of patients with gastric cancer. CEACAM1-S-dominant expression in gastric cancer tissue indicates better survival from patients with gastric cancer than patients without CEACAM1 expression and with CEACAM1-L dominance. CEACAM1 may become a biomarker for patients with gastric cancer with peritoneal dissemination. CEACAM1 expression, especially CEACAM1-S expression, may be a therapeutic target for peritoneal dissemination of patients with gastric cancer. Further analysis of CEACAM1 on gastric cancer cells may shed light on the diagnosis and treatment for patients with gastric cancer.

## Methods

### Patients

A total of 235 patients without distant metastasis who received radical surgery at Wakayama Medical University Hospital (WMUH) between January 2005 from December 2006 were enrolled in this study. Surgical specimens were submitted for immunohistochemistry. They included 128 stage I, 59 stage II, 48 stage III, and 0 stage IV gastric carcinomas based on TNM classification. The mean age of the patients was 66.6 years, and there were 170 male and 65 female subjects. The follow-up period was five years. Stage II and III patients without mucosal cancer received S-1 (oral fluoropyrimidine)-based postoperative chemotherapy, stage I patients received no chemotherapy. The present study was approved by the Human Ethics Review Committee of Wakayama Medical University (Approval Number 1656). We carried out all experiments in compliance with declaration of Helsinki, the guidelines for ethical principles for medical research involving Human Subjects, and the ethical guidelines of Wakayama Medical University. Informed consent was obtained in the form of opt-out on the web page of Wakayama Medical University.

### Immunohistochemistry

Immunohistological staining using the streptavidin-biotin method was performed as described before^7–9^. Tissue sections with 4 μm thickness were created from formalin-fixed paraffin-embedded blocks. Sections were deparaffinized and autoclaved at 121 °C for 10 min in 0.1 M citrate buffer (pH 6.0). Endogenous peroxidase activities were blocked by incubation in 3% hydrogen peroxide for 5 min at room temperature. Thos glass slides were washed with PBS solution and incubated with serum-free protein blocking solution (X0909, DAKO, USA) for 10 min at room temperature. Then, the sections were incubated with the primary antibodies, mouse anti-human CEACAM1 monoclonal antibody 4D1/C2 (MABT65, Merc, dilution 1:200) or rabbit anti-human CEACAM1-long-specific polyclonal antibodies (dilution 1:500) or mouse anti-human CEACAM1-short-specific polyclonal antibodies (dilution 1:500), both antibodies were created by John E. Shively, and their specificity are confirmed by western blotting^7^, for 18 h at 4 °C. A biotinylated secondary antibody and peroxidase-conjugated streptavidin from the Histofine MAX-PO (M) kit (Nichirei, Japan) were applied for 30 min at room temperature. Finally, the sections were incubated in 3′3-diaminobenzidine for 10 min, followed by hematoxylin counterstaining and slide mounting.

### Evaluation of immunohistochemical staining

Expression status was evaluated by scoring both the intensity and the range of staining as described before^8,12^. The intensity of the staining was scored as 0 (negative), 1 (weak), 2 (moderate), 3 (strong); the range of the general staining intensity in the tissue was scored as 0 (0–5%), 1 (6–33%), 2 (34–66%), or 3 (67–100%). The ranges of staining and intensity scores were then added to obtain a total score^12^. All specimens were twice blindly reviewed by three individuals. If discrepancies arose, the specimens were viewed with a multi-head microscope and discussed in order to achieve a consensus.

### Cell lines

Human gastric cancer cell lines were used in the present study. NUGC3 (undifferentiated type) and MKN7 (differentiated type) cells were purchased from Japanese Collection of Research Biosources (Tokyo, Japan). NUGC3 and MKN7 cells were maintained in RPMI-1640 (Wako) medium containing 10% fetal bovine serum (FBS) (Gibco), 100 U/ml of penicillin G, and 100 μg/ml of streptomycin at 37 °C in a humidified 5% CO_2_ atmosphere.

### Transfection and RNA interference

Transfection and RNA interference techniques were previously described^7–9,15,20^. CEACAM1-4L or -4S cDNA was cloned into the pH-β actin vector^23^, and was mixed with 1 μL of Lipofectamine 3000 (Invitrogen) to a final volume of 100 μL of Opti-MEM medium, and was added to NUGC3 cells grown to 80% confluence in 24-well plates. Forty-eight hours after transfection, a G418 solution (Roche, Basel, Switzerland) was added. The stably transfected cells were maintained in the culture media described above. Short hairpin RNA (shRNA) plasmids designed to target CEACAM1 were synthesized by SABiosciences (Frederick, MD, USA), as described before^7^. Each plasmid (0.8 μg) was transfected into MKN7 cells as described above. The expression level of CEACAM1 of the stably transfected cell lines was evaluated by reverse transcription-polymerase chain reaction (Fig. 5A)

### Invasion assay

Invasion assay was conducted using Biocoat Matrigel invasion chambers (BD Biosciences), techniques were previously described^7,8^. Each measurement was performed in quadruplicate. The percentage invasion was calculated as the ratio of the number of cells through the Matrigel membrane to the number of cells migrating through the control membrane.

### Matrigel Culture

Matrigel 3D culture were previously described^20^. Matrigel (200 µl, BD Biosciences, Bedford, MA) was plated on 24-well plates and allowed to solidify for 15 min at 37 °C. After the Matrigel solidified, 0.5 ml of the cell suspension in the culture medium containing 2 × 10^4^/ml was directly seeded into each well and incubated at 37 °C in a 5% humidified CO2 atmosphere for 2 h. After incubation, Matrigel (200 µl, 50% concentration) was added to the upper layer and allowed to solidify for 15 min at 37 °C. Complete culture medium was then added, and the plates were incubated at 37 °C in a 5% humidified CO2 atmosphere. During culture, cells were regularly monitored for cell growth and lumen formation, which was scored by the presence or absence of lumen. Culture medium was changed every three days until day 15.

### *In vivo* tumor mouse model

Six-week-old NOD.CB17-*Prkdc*^*scid*^/J (NOD SCID) mice were purchased from Charles River Laboratories Japan Inc., Shiga, Japan. All animal experiments were conducted in accordance with the institutional guidelines of the National Institutes of Biomedical Innovation, Health and Nutrition. NUGC3 cells (100 µl of 5 × 10^6^ cells/ml in PBS) transfected with CEACAM1-vector control, CEACAM1-4L, or CEACAM1-4S were inoculated subcutaneously into the back of mice. The tumor size was measured twice a week by length (L), width (W), and height (H). Tumor volume (V) was calculated as V = (L × W × H)/2. All animal experiments were performed in compliance with the Japanese Government’s Animal Protection and Management Law (no. 105) and Standards Relating to the Care and Management of Laboratory Animals and Relief of Pain (no. 88), and the guidelines for animal experiments of Wakayama Medical University. This study was approved by The Committee of Animal Experiments (Approval Number 878) and the Committee of Gene Recombination (Approval Number 29–13) of Wakayama Medical University.

### Statistical analysis

Univariate cox regression for the overall survival was used for statistical analyses clinicopathological factor. Those factors were also analyzed by multivariate cox regression, and odds ratio with a 95% confidence interval was calculated for each factor. Kaplan-Meier method was demonstrated to estimate overall survival. The log-rank test was employed to determine the statistical significance of those data. All data were presented as mean ± SD. *P*-value < 0.05 was considered to be statistically significant. Statistical calculations were performed using SPSS software program ver. 18.0 (SPSS, Chicago, IL).

## Supplementary information

Supplementary Info

## References

[1] Thompson JA, Grunert F, Zimmermann W (1991). Carcinoembryonic antigen gene family: molecular biology and clinical perspectives. J Clin Lab Anal.

[2] Riethdorf L (1997). Differential expression of CD66a (BGP), a cell adhesion molecule of the carcinoembryonic antigen family, in benign, premalignant, and malignant lesions of the human mammary gland. J Histochem Cytochem.

[3] Luo W (1999). Tumor-suppressive activity of CD66a in prostate cancer. Cancer Gene Ther.

[4] Bamberger AM (1998). Dysregulated expression of CD66a (BGP, C-CAM), an adhesion molecule of the CEA family, in endometrial cancer. The American journal of pathology.

[5] Thies A (2002). CEACAM1 expression in cutaneous malignant melanoma predicts the development of metastatic disease. J Clin Oncol.

[6] Laack E (2002). Expression of CEACAM1 in adenocarcinoma of the lung: a factor of independent prognostic significance. J Clin Oncol.

[7] Ieda J (2011). Re-expression of CEACAM1 long cytoplasmic domain isoform is associated with invasion and migration of colorectal cancer. *International journal of cancer*. Journal international du cancer.

[8] Kiriyama S (2014). CEACAM1 long cytoplasmic domain isoform is associated with invasion and recurrence of hepatocellular carcinoma. Annals of surgical oncology.

[9] Yamaguchi S (2017). CEACAM1 is associated with recurrence after hepatectomy for colorectal liver metastasis. The Journal of surgical research.

[10] Zhou CJ (2009). The different expression of carcinoembryonic antigen-related cell adhesion molecule 1 (CEACAM1) and possible roles in gastric carcinomas. Pathol Res Pract.

[11] Shi JF, Xu SX, He P, Xi ZH (2014). Expression of carcinoembryonic antigen-related cell adhesion molecule 1(CEACAM1) and its correlation with angiogenesis in gastric cancer. Pathol Res Pract.

[12] Guo JQ (2012). Different expression patterns of CEACAM1 and its impacts on angiogenesis in gastric nonneoplastic and neoplastic lesions. Annals of surgical oncology.

[13] Yokoyama S, Chen CJ, Nguyen T, Shively JE (2007). Role of CEACAM1 isoforms in an *in vivo* model of mammary morphogenesis: mutational analysis of the cytoplasmic domain of CEACAM1-4S reveals key residues involved in lumen formation. Oncogene.

[14] Yokoyama S (2015). P4H9-detected molecule expression on spindle-shaped fibroblasts indicates malignant phenotype of colorectal cancer. British journal of cancer.

[15] Yamamoto N (2015). CEACAM1 and hollow spheroid formation modulate the chemosensitivity of colorectal cancer to 5-fluorouracil. Cancer chemotherapy and pharmacology.

[16] Neumaier M, Paululat S, Chan A, Matthaes P, Wagener C (1993). Biliary glycoprotein, a potential human cell adhesion molecule, is down-regulated in colorectal carcinomas. Proc Natl Acad Sci USA.

[17] Huang J (1998). Expression of biliary glycoprotein (CD66a) in normal and malignant breast epithelial cells. Anticancer Res.

[18] Huang J, Hardy JD, Sun Y, Shively JE (1999). Essential role of biliary glycoprotein (CD66a) in morphogenesis of the human mammary epithelial cell line MCF10F. J Cell Sci.

[19] Kirshner J, Chen CJ, Liu P, Huang J, Shively JE (2003). CEACAM1-4S, a cell-cell adhesion molecule, mediates apoptosis and reverts mammary carcinoma cells to a normal morphogenic phenotype in a 3D culture. Proc Natl Acad Sci USA.

[20] Tamura K (2011). Hollow spheroids beyond the invasive margin indicate the malignant potential of colorectal cancer. BMJ open.

[21] Cruz PV, Wakai T, Shirai Y, Yokoyama N, Hatakeyama K (2005). Loss of carcinoembryonic antigen-related cell adhesion molecule 1 expression is an adverse prognostic factor in hepatocellular carcinoma. Cancer.

[22] Turbide C, Kunath T, Daniels E, Beauchemin N (1997). Optimal ratios of biliary glycoprotein isoforms required for inhibition of colonic tumor cell growth. Cancer Res.

[23] Gunning P, Leavitt J, Muscat G, Ng SY, Kedes L (1987). A human beta-actin expression vector system directs high-level accumulation of antisense transcripts. Proc Natl Acad Sci USA.

