# Supplementary material for: Loss of CEACAM1 is associated with poor prognosis and peritoneal dissemination of patients with gastric cancer
Source: Sci Rep. 2019 Sep 3;9:12702. doi: 10.1038/s41598-019-49230-w (PMC6722051; doi:10.1038/s41598-019-49230-w)

Loss of CEACAM1 is associated with poor prognosis and peritoneal dissemination of patients with gastric cancer

Akihiro Takeuchi, MD[1], Shozo Yokoyama, MD, PhD[1]*, Mikihito Nakamori, MD, PhD[1], Masaki Nakamura, MD, PhD[1], Toshiyasu Ojima, MD, PhD[1], Shunsuke Yamaguchi, MD, PhD[1], Yasuyuki Mitani, MD[1], John E. Shively, PhD[2], and Hiroki Yamaue, MD, PhD[1]

[1] Second Department of Surgery, School of Medicine, Wakayama Medical University, Wakayama, 641-0045, Japan
[2] Department of Molecular Immunology, Beckman Research Institute of the City of Hope, Duarte, CA, 91010 USA

Figure 5A

NUGC3

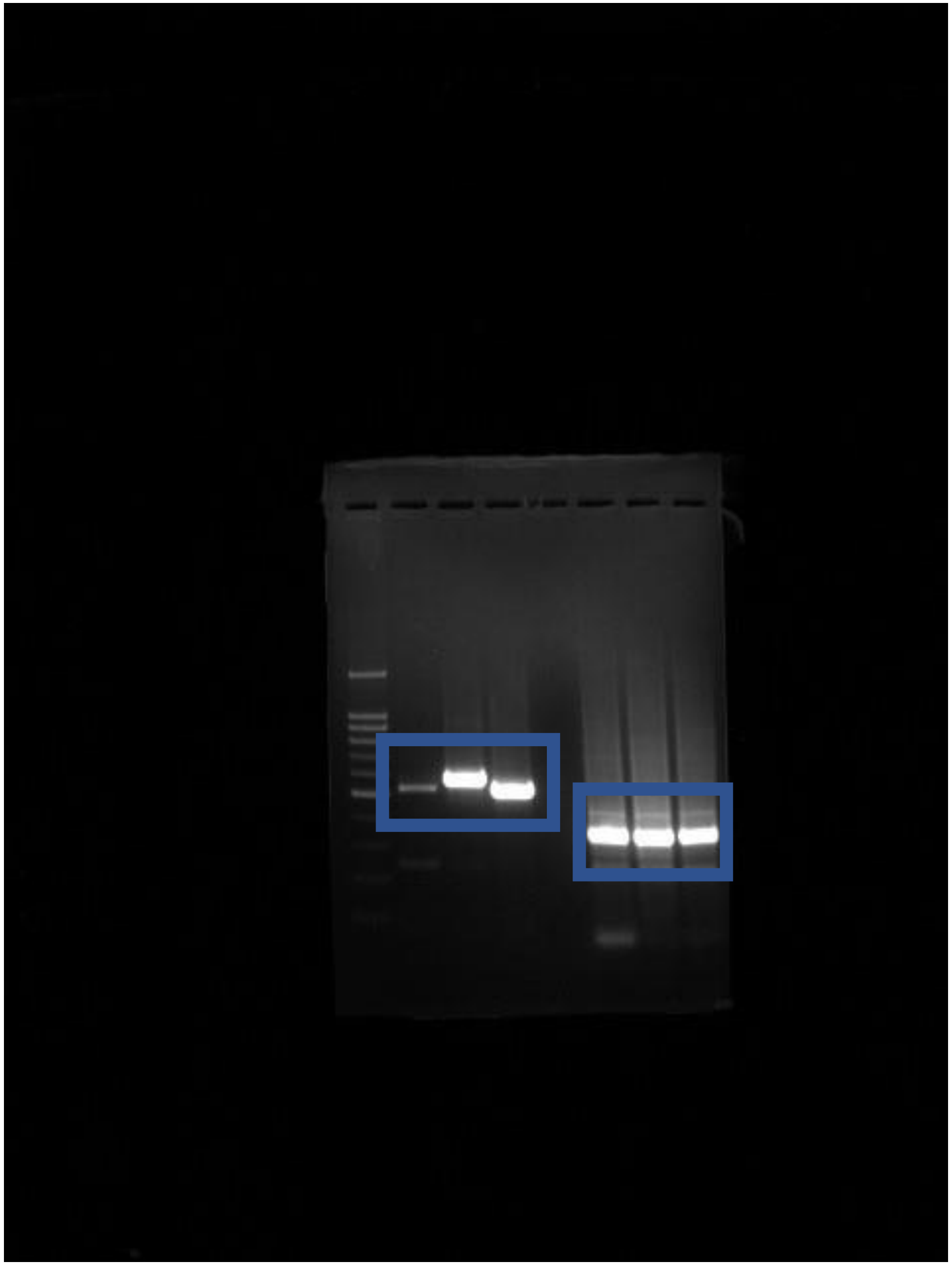

Figure 5A

MKN7

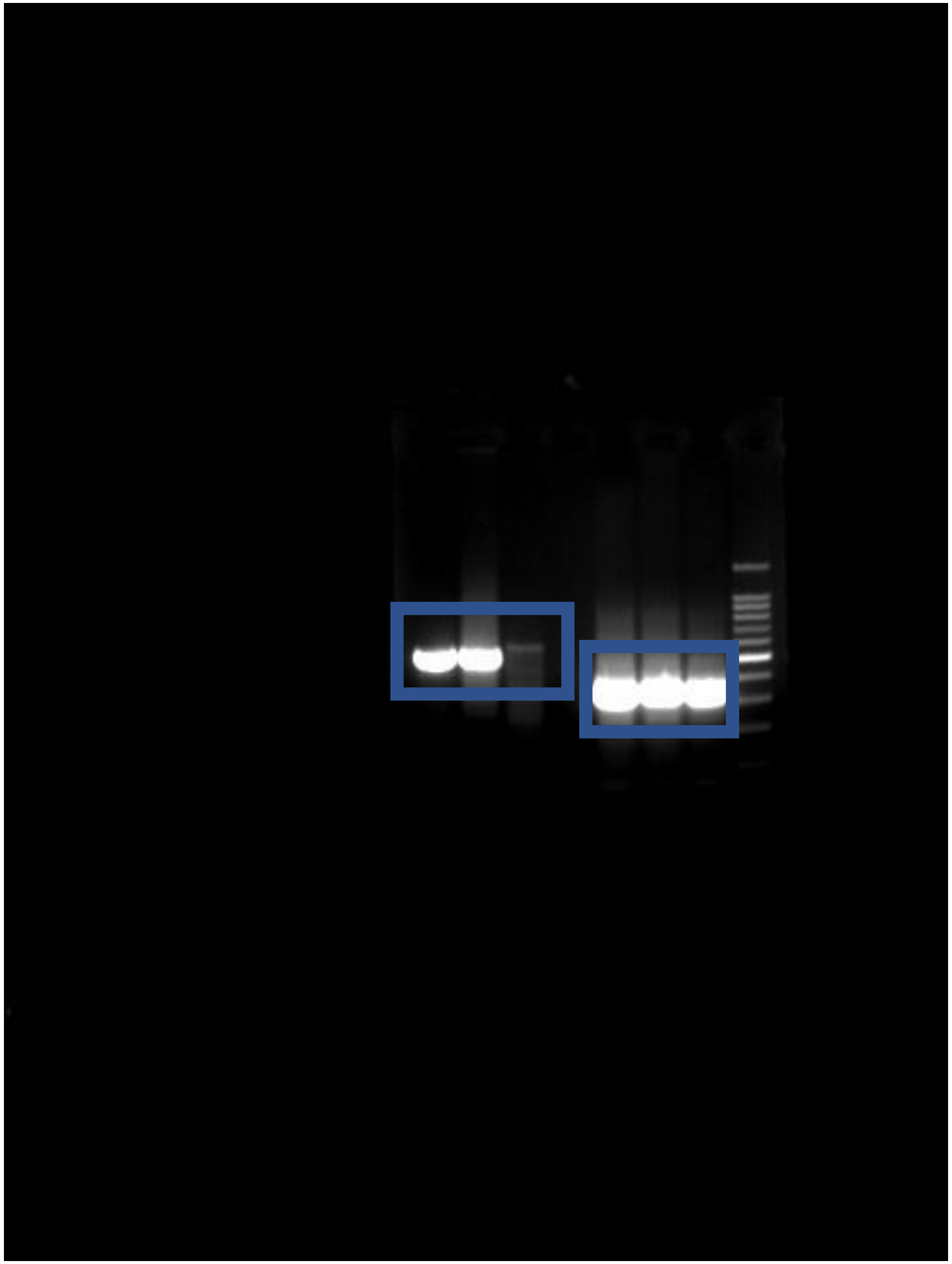

Supplement: Supplementary file 1 — Supplementary Info [file 41598_2019_49230_MOESM1_ESM.pdf]
